# Regulation of Metabolic Activity by p53

**DOI:** 10.3390/metabo7020021

**Published:** 2017-05-20

**Authors:** Jessica Flöter, Irem Kaymak, Almut Schulze

**Affiliations:** 1 Department of Biochemistry and Molecular Biology, Theodor-Boveri-Institute, Biocenter, Am Hubland, 97074 Würzburg, Germany; j.schmitt@uni-wuerzburg.de (J.F.); irem.kaymak@uni-wuerzburg.de (I.K.); 2 Comprehensive Cancer Center Mainfranken, Josef-Schneider-Strasse 6, 97080 Würzburg, Germany

**Keywords:** cancer metabolism, p53 tumour suppressor, fatty acid metabolism, cholesterol, microenvironment

## Abstract

Metabolic reprogramming in cancer cells is controlled by the activation of multiple oncogenic signalling pathways in order to promote macromolecule biosynthesis during rapid proliferation. Cancer cells also need to adapt their metabolism to survive and multiply under the metabolically compromised conditions provided by the tumour microenvironment. The tumour suppressor p53 interacts with the metabolic network at multiple nodes, mostly to reduce anabolic metabolism and promote preservation of cellular energy under conditions of nutrient restriction. Inactivation of this tumour suppressor by deletion or mutation is a frequent event in human cancer. While loss of p53 function lifts an important barrier to cancer development by deleting cell cycle and apoptosis checkpoints, it also removes a crucial regulatory mechanism and can render cancer cells highly sensitive to metabolic perturbation. In this review, we will summarise the major concepts of metabolic regulation by p53 and explore how this knowledge can be used to selectively target p53 deficient cancer cells in the context of the tumour microenvironment.

## 1. p53 and Tumour Suppression

*TP53* was identified in 1979 [1] and was initially believed to be a proto-oncogene, due to the high frequency of point mutations found in cancers (reviewed in [2]). However, 10 years later it was realised that loss or mutation of *TP53* inactivates one of the most important tumour suppressors [3,4].

The *TP53* gene encodes a crucial transcription factor, which controls the expression of genes involved in cell cycle regulation, apoptosis and DNA repair. Levels of the p53 protein are induced after DNA damage, oncogene activation and telomere erosion as well as in response to loss of stromal support, nutrient and oxygen deprivation, induction of ribosomal and endoplasmatic reticulum stress and viral infection [5,6,7,8,9]. The low basal expression of p53 in unstressed cells is maintained by MDM2/HDM2 (mouse/human double minute 2 homolog) and MDM4 (also called MDMX) [10]. MDM2 is an E3 ubiquitin ligase that induces ubiquitylation and degradation of p53, thereby preventing induction of p53 target genes [11,12]. In response to DNA damage or oncogenic stress, p53 is activated through post-translational modifications, such as acetylation or phosphorylation, which prevent its interaction with MDM2 and lead to an increased stability of p53 [11,12]. Transient cell cycle arrest induced by p53 allows DNA repair and supports genome stability and cell survival [13,14]. However, strong or sustained activation of p53 leads to the induction of apoptotic cell death by upregulating PUMA, NOXA or BAX [15,16] or senescence by upregulation of p21, thereby providing a barrier towards cell transformation and tumour development [2,8].

It is therefore not surprising that 50% of all human tumours carry genetic alterations that lead to the inactivation of the p53 pathway. Mostly, these alterations are missense mutations in the coding region of the *TP53* gene, but this varies among different tumour types [17,18]. P53 mutations are mainly found in solid tumours and occur at high frequency in inflammation-associated cancers [19,20,21,22]. Many p53 mutations cause conformational changes of the DNA binding domain of the p53 protein, leading to reduced binding of p53 to the promoters of its target genes [23]. Importantly, as p53 functions as a tetramer [24], the presence of mutant p53 in cancer cells has a dominant negative effect on wild type p53 function even in heterozygous cells. Moreover, since mutant p53 cannot activate the expression of its negative regulator MDM2, mutant p53 protein is stabilised [25] and can exert additional tumour promoting functions [26]. In general, loss of p53 function causes resistance to DNA damage and prevents apoptosis or senescence in cancer cells [27,28,29].

**Figure 1:**
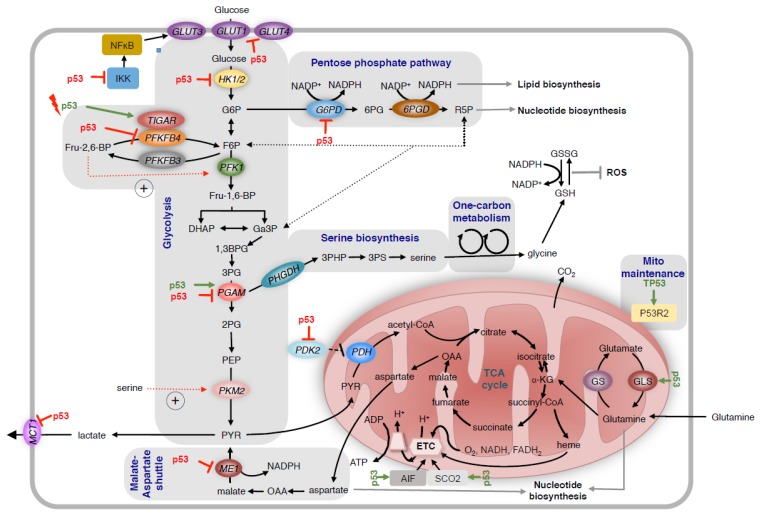
Regulation of glycolysis and mitochondrial metabolism by p53.

Tumour development is accompanied by changes in cellular metabolic activity, which allows cancer cells to grow and proliferate under adverse conditions. The influence of p53 on cellular metabolism is complex and involves multiples nodes of regulation (summarised in Figure 1). p53 changes the activity of multiple metabolic pathways, including glycolysis, mitochondrial oxidative phosphorylation and fatty acid synthesis via transcriptional and non-transcriptional regulation. In addition, p53 governs the adaptation of cancer cells to nutrient and oxygen deprivation, which is crucial for the survival under the metabolically compromised conditions shaped by the tumour microenvironment. Importantly, it has been shown that the regulation of metabolic activity is essential to the tumour suppressive function of p53 [30].

p53 regulates glycolysis and mitochondrial metabolism through multiple mechanisms. It reduces glucose uptake by directly repressing the transcription of genes coding for the glucose transporters GLUT1 and GLUT4 and by indirectly repressing GLUT3. p53 also reduces expression of *HK2*, which controls the production of G6P. In response to acute activation by DNA damage, p53 induces the expression of *TIGAR*. This reduces levels of Fru-2,6-BP and decreases the activity of PFK1, leading to the diversion of G6P into the oxidative arm of the PPP. In contrast, loss of p53 increases the expression of PFKFB4, which also reduces the production of Fru-2,6-BP and promotes pentose phosphate pathway activity in p53 deficient cancer cells. P53 also alters the activity of the pentose phosphate pathway by directly inhibiting the activity of G6PD. Depending on the tissue, p53 either increases or decreases the expression of PGAM. Suppression of *MCT1* by p53 reduces lactate secretion. Conversely, inhibition of PDK2 expression by p53 results in increased conversion of pyruvate to mitochondrial acetyl-CoA. To regulate mitochondrial metabolism, p53 enhances the expression of *SCO2* and *AIF*, two factors controlling the assembly of complexes of the electron transport chain (ETC). P53 also controls mitochondrial maintenance through inducing *P53R2*. Increased expression of GLS2 promotes glutamine dependent anaplerosis of the TCA cycle. HK2 = hexokinase 2; G6P = glucose-6-phosphate; TIGAR = *TP53* induced glycolysis regulatory phosphatase; Fru-2,6-BP = fructose-2,6-bisphosphate; PFKFB4 = phosphofructokinase-2/fructose-2-bisphosphatases; PGAM = phosphoglycerate mutase; MCT1 = monocarboxylate transporter 1; SCO2 = synthesis of cytochrome c oxidase assembly 2; AIF = apoptosis-inducing factor; GLS2 = glutaminase 2; PDK2 = pyruvate dehydrogenase kinase 2; PPP = pentose phosphate pathway; ETC = electron transport chain; TCA = tricarboxylic acid cycle; ME1 = malic enzyme 1.

## 2. Regulation of Glycolysis and the Pentose Phosphate Pathway

One of the hallmarks of cancer is the altered metabolic activity of cancer cells [31] and many cancer cells rely on glycolysis as the predominant source of ATP production, even in the presence of oxygen. This metabolic reprogramming leads to increased glucose uptake and lactate production and is generally known as aerobic glycolysis or the “Warburg effect” [32]. Initially, this seems to be a paradox as oxidative phosphorylation produces substantially more ATP than glycolysis. However, it is now established that ATP production is not a rate-limiting process for cell proliferation and that increased glucose uptake allows cancer cells to generate glycolytic intermediates for essential biosynthetic processes, such as the synthesis of riboses for nucleotide biosynthesis [33].

The ability of cancer cells to reprogram their metabolism towards aerobic glycolysis is counteracted by wild type p53 through multiple mechanisms (Figure 1). Wild type p53 reduces the expression of the glucose transporters GLUT1 and GLUT4 through direct transcriptional repression [34]. Furthermore, p53 blocks the expression of GLUT3 by interfering with the activity of IκB kinases α and β (IKKα/β) and inhibiting nuclear factor kappa B1 (NF-κB) [35]. Similarly, the promoter of the gene coding for hexokinase 2 (HK2) contains several p53 binding sites and is repressed by wild type p53 [36]. Together, glucose transporters and hexokinases control the levels of glucose-6-phosphate (G6P), a central metabolite that is directed into glycolysis, glycogen synthesis and the pentose phosphate pathway (PPP).

In glycolysis, G6P is first converted to fructose-6-phosphate (F6P), the substrate of phosphofructokinase 1 (PFK1). PFK1 then catalyses the conversion of F6P to fructose-1,6-bisphosphate, the rate-limiting step of glycolysis, and the activity of this enzyme is tightly regulated. High cellular energy load, indicated by a high ATP/ADP ratio or high amounts of cytoplasmic citrate, inhibits the activity of PFK1 and blocks degradation of glucose. In addition, the activity of PFK1 is controlled by fructose-2,6-bisphosphate (Fru-2,6-BP), which is generated from F6P by the phosphofructokinase-2/fructose-2-bisphosphatases (PFK/FBPases). PFK/FBPases are bi-functional enzymes with two catalytic centres, a kinase and a phosphatase activity, that act independently to control the amount of Fru-2,6-BP [37,38]. The human genome encodes four PFK/FBPase proteins (PFKFB1-4), which differ in their tissue specific expression and relative activity of their respective kinase and phosphatase domains. Notably, PFKFB3, which has the highest ratio of the relative kinase to phosphatase activity, is highly expressed in many cancers and can contribute to the induction of the Warburg effect [39].

Interestingly, the *TP53* induced glycolysis regulatory phosphatase (TIGAR) shows sequence homology to the phosphatase domain of PFKFB proteins [40]. TIGAR is induced upon acute activation of p53 in response to DNA damage. This blocks PFK1 activity by reducing Fru-2,6-BP levels. As a consequence, G6P is redirected into the PPP to support enhanced ribose production for nucleotide synthesis during DNA repair [40,41]. TIGAR also limits autophagy by preventing the accumulation of cellular reactive oxygen species (ROS) [42]. Furthermore, p53 can be activated by inhibition of the PPP, indicating a negative feedback loop to restore PPP activity through p53 and TIGAR to protect cells from ROS-associated damage [43,44]. However, the exact role of TIGAR as a regulator of glycolysis is not entirely resolved. In vitro studies showed that TIGAR preferentially dephosphorylates 2,3-bisphosphoglycerate rather than Fru-2,6-BP [45]. Moreover, TIGAR is required for intestinal regeneration and tumourigenesis [46], but is upregulated in cancer through mechanism independent of p53 or p73 [47], suggesting possible additional functions of this enzyme.

In contrast to TIGAR, PFKFB4, the testis isoenzyme of PFK/FBPases, is downregulated by p53. It has been shown that p53 binds to the *PFKFB4* promoter, leading to transcriptional repression via histone deacetylases [48]. PFKFB4 was previously shown to be essential for the survival of prostate cancer cells [49] and glioma stem-like cells [50]. Depletion of PFKFB4 from p53 deficient cells increases Fru-2,6-BP levels, thereby enhancing glycolytic flux and depleting metabolites from the PPP [48]. As a consequence, NADPH levels are lowered, leading to oxidative stress, reduced cell viability and decreased tumour growth, suggesting that PFKFB4 supports survival of p53-deficient cancer cells.

The activity of the PPP is also directly controlled by p53 through interaction with glucose-6-phosphate dehydrogenase (G6PD), the first and rate-limiting enzyme of this pathway. Binding of p53 to G6PD prevents formation of the active enzyme dimer, leading to a decrease in enzyme activity. Mutants of p53, often found in tumours, lack this G6PD-inhibitory activity. This leads to the activation of metabolite entry into the PPP and directs glucose towards biosynthesis [51]. Together, these results demonstrate that cancer cells need to accurately control the relative metabolite flux between glycolysis and the PPP to generate glycolytic intermediates but also maintain NADPH regeneration and nucleotide biosynthesis.

The seemingly opposing effects of p53 on PPP activity can potentially be explained by the context within which p53 exerts its function. While acute activation of p53 in response to DNA damage temporarily increases the cellular demand for NADPH and nucleotides for DNA repair, loss or mutation of p53 in cancer also enhances biosynthetic demand due to increased proliferation and therefore requires similar metabolic adaptations. This deregulation of metabolic activity renders p53 deficient cancer cells highly dependent on NADPH regenerating processes and makes them highly susceptible to perturbation. As a consequence, disrupting metabolic control mechanisms that maintain the balance of metabolite flux between glycolysis and PPP should selectively impair the survival of p53 deficient cancer cells and could be a strategy for therapeutic intervention.

## 3. Regulation of Serine Metabolism

Glycolysis also provides essential intermediates for biosynthetic pathways, including the synthesis of serine and glycine. p53 drives ubiquitination and inactivation of phosphoglycerate mutase 1 (PGAM1) [52], an enzyme of the glycolytic pathway that converts 3-phosphoglycerate (3PG) to 2-phosphoglycerate (2PG). Consequently, mutation of p53 in fibroblasts leads to an increased glycolytic rate but decreases the flux of metabolites into serine and glycine biosynthesis. 3PG is also an allosteric inhibitor of 6-phosphogluconate dehydrogenase (6PGD), the second NADPH producing enzyme of the PPP. Downregulation of PGAM by p53 leads to the accumulation of this metabolite thereby further inhibiting PPP activity [53]. However, p53 was also shown to increase the expression of phosphoglycerate mutase 2 (PGAM2) in cardiocytes [54], suggesting that the control of this enzymatic activity is tissue specific.

It has also been demonstrated that high PGAM1 expression in cancer cells maintains serine biosynthesis through activation of phosphoglycerate dehydrogenase (PHGDH), the first enzyme of the serine biosynthesis pathway, which is allosterically activated by 2PG [53]. Furthermore, serine binds to and activates the M2 isoform of pyruvate kinase (PKM2). Inhibition of PKM2 in the absence of serine results in the accumulation of metabolic intermediates that can be used for *de novo* serine biosynthesis [55,56] and also promotes the activity of the oxidative branch of the PPP for NADPH regeneration and antioxidant production [57]. In the absence of exogenous serine, glycine is converted into serine by consuming one-carbon units and thus nucleotide synthesis is inhibited [58]. Serine availability therefore represents a crucial node in the metabolism of tumour cells.

Interestingly, p53 deficient cancer cells are highly susceptible to serine starvation [59]. Under these conditions, induction of *de novo* serine synthesis competes for substrates with other metabolic processes, most importantly nucleotide biosynthesis and the production of glutathione (GSH) [59]. Cancer cells deficient for p53 are unable to induce cell cycle arrest in response to serine starvation. This keeps the demand for nucleotide synthesis high and reduces the production of GSH, leading to oxidative stress and cell death [59]. The serine biosynthesis pathway also intersects with the folate and methionine cycles, which produce methyl-groups for nucleotide synthesis as well as for histone and DNA methylation [60]. During methionine starvation, serine provides one-carbon units to recycle homocysteine to methionine [61]. In addition, in colorectal cancer cells serine-dependent *de novo* ATP synthesis supports the conversion of methionine to S-adenosyl methionine (SAM), the primary cellular methyl donor [61]. However, in a genetic model of pancreatic cancer driven by activation of Kras together with either loss or mutation of p53, combined removal of serine and glycine from the diet had no effect on tumour growth [62]. This indicates that the importance of these non-essential amino acids depends on tumour type.

## 4. Regulation of Mitochondrial Metabolism

Mitochondrial metabolism is an important source of ATP generation but also provides intermediates for multiple biosynthetic reactions. It has been shown that p53 regulates mitochondrial DNA copy number and mitochondrial mass [63,64] and promotes oxidative phosphorylation via transcriptional activation of *p53R2* (*RRM2B*), a subunit of ribonucleotide reductase [64,65]. In addition, p53 transcriptionally increases the expression of the cytochrome c oxidase assembly 2 protein (*SCO2*), which is needed for the assembly of the cytochrome c oxidase complex (complex IV in the electron transport chain) [66] and apoptosis-inducing factor (*AIF*) [67], a mitochondrial flavoprotein required to maintain mitochondrial complex I activity. In addition, p53 regulates the COXII subunit of complex IV, which is encoded by the mitochondrial genome, through a post-transcriptional mechanism [68].

Another crucial node in p53 dependent metabolic control is the regulation of pyruvate metabolism. p53 transcriptionally represses pyruvate dehydrogenase kinase-2 (*PDK2*), which phosphorylates and inhibits the pyruvate dehydrogenase (PDH) activity [69]. Through this mechanism, p53 promotes the conversion of pyruvate into acetyl-CoA for entry into the tricarboxylic acid (TCA) cycle and positively regulates glucose oxidation [69]. Moreover, p53 suppresses the expression of the lactate/proton symporter monocarboxylate transporter 1 (*MCT1*), thereby reducing the ability of cells to regenerate NAD+ through the conversion of pyruvate to lactate [70]. As a consequence, cells that lack p53 generate less ATP through oxidative phosphorylation compared to p53 proficient cells [71].

In addition to glucose-derived pyruvate, the TCA cycle can also be fuelled by glutamine via α-ketoglutarate dependent anaplerosis. p53 regulates glutaminolysis by binding to p53 consensus DNA-binding elements in the promoter of the gene coding for glutaminase 2 (GLS2), a mitochondrial enzyme that catalyses the hydrolysis of glutamine to glutamate. Increased GLS2 expression enhances mitochondrial respiration, ATP generation and glutathione (GSH) production and decreases cellular ROS levels [72,73], discussed in more detail below.

## 5. Regulation of Oxidative Stress

Oxidative stress has been linked to DNA damage and karyotype instability and was shown to be essential for tumour development in p53 deficient mice [44]. However, different studies indicate that p53 can have a positive or negative effect on ROS levels, depending on cellular context. Activation of p53 by DNA damage or other stresses induces the expression of genes encoding pro-oxidant enzymes, such as the p53-induced protein PIG3 (TP53I3) [74], the pro-apoptotic factors PUMA (BBC3) [75,76] and NOXA (PMAIP1) [77], and the proline-oxidase (PRODH) [78]. Increased expression of factors that promote mitochondrial respiration can also enhance oxidative stress in response to p53 activation [66]. Conversely, p53 reduces the expression of pro-oxidant genes, such as nitric oxide synthase (NOS2) [79] or cyclooxygenase 2 (COX2) [80].

Antioxidant factors that prevent or remove cellular ROS are also modulated by p53. p53 increases the expression of antioxidant systems, for example the stress-inducible sestrin proteins [81,82] or the p53-inducible nuclear protein 1 (*TP53INP1)* [83]. Furthermore, the p53 target p21 directly interacts with the nuclear factor NRF2 (NFE2L2), leading to upregulation of the antioxidant response [84]. p53 also induces the expression of Parkin (*PARK2*), a component of an E3 ubiquitin ligase complex and regulator of energy metabolism and antioxidant defense [85].

On the other hand, p53 blocks a number of important cellular antioxidant pathways. For example, p53 reduces the activity of superoxide dismutase 2 (SOD2), the main enzyme responsible for the removal of superoxide from the mitochondrial matrix [86]. An important metabolite for the removal of cytoplasmic ROS is NADPH, which is required for the regeneration of the antioxidants GSH and thioredoxin (TXN). As already mentioned above, inhibition of G6PD activity by p53 limits NADPH production by the oxidative PPP [51]. Moreover, p53 reduces the expression of malic enzymes 1 and 2 (*ME1* and *ME2*), which also contribute to cellular NADPH production [87]. p53 also reduces the function of the cystine/glutamate antiporter (system Xc-) by negatively regulating the expression of the solute carrier family 7 member 11 (*SLC7A11*) [88]. This limits the availability of cysteine for GSH synthesis, leading to the accumulation of lipid peroxides and induction of ferroptosis, an iron-dependent form of cell death [89]. Indeed, induction of ferroptosis through downregulation of SLC7A11 is an essential part of the tumour suppressor function of p53 [88].

## 6. Regulation of Lipid Metabolism

Lipid metabolism is tightly regulated in cancer cells [90,91]. Many cancers show enhanced rates of fatty acid biosynthesis as part of their overall increase in anabolic metabolism during rapid proliferation [92]. Some cancers also increase the degradation of fatty acids, which can contribute to ATP production via mitochondrial β-oxidation or supply metabolic intermediates, including acetyl-CoA or citrate [93].

**Figure 2:**
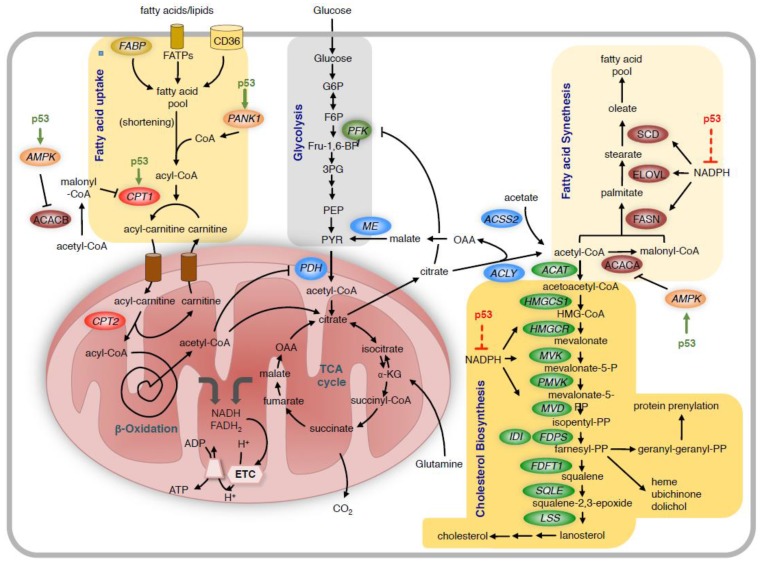
Regulation of lipid metabolism by p53.

p53 exerts its effect on lipid metabolism through several mechanisms (Figure 2). In line with its role in blocking anabolic metabolism, p53 decreases fatty acid synthesis but enhances fatty acid degradation [94]. As outlined above, p53 reduces global cellular anabolic metabolism by limiting NADPH production, which is an essential cofactor for many biosynthetic reactions including fatty acid synthesis. p53 also induces the AMP activated protein kinase (*AMPK*), which blocks fatty acid biosynthesis by phosphorylation of acetyl-CoA carboxylases 1 and 2 (ACACA and ACACB) [95].

Fatty acids are taken up by the cells through receptor and non-receptor mediated transport. Unless prior shortening of very long chain fatty acids in peroxysomes is required, medium and short chain fatty acids are coupled to coenzyme A (CoA) in the cytoplasm and then transferred to carnitin by CPT1 for transport across the mitochondrial membrane. In the mitochondria, the acyl-chain is transferred back to CoA and then undergoes repeated shortening by the β-oxidation process. This produces NADH and FADH_2_, which provide electrons to the ETC, and acetyl-CoA, which enters the TCA cycle. P53 promotes fatty acid metabolism by increasing the expression of *CPT1C*. p53 also activates AMPK, which phosphorylates and inhibits ACACB, leading to reduced production of malonyl-CoA, thereby releasing the inhibition of CPT1. High levels of mitochondrial acetyl-CoA also block PDH and reduce the use of glucose-derived pyruvate in the TCA cycle. The TCA cycle also provides citrate, which is transported out of the mitochondria and is converted to acetyl-CoA and oxaloacetate (OAA) by ACLY. Another source of acetyl-CoA is the direct conversion of acetate to acetyl-CoA by acetyl-CoA synthetase 2 (ACSS2). Acetyl-CoA is the substrate for the synthesis of fatty acids and cholesterol. For fatty acid synthesis, acetyl-CoA is first converted to malonyl-CoA by ACACA, and the two metabolites are then sequentially condensed by the multifunctional enzyme FASN to form the fatty acid palmitate. This is then further elongated and desaturated to contribute to the cellular fatty acid pool. For cholesterol biosynthesis, acetyl-CoA is first converted to acetoacetyl-CoA, which enters the mevalonate pathway. In addition to cholesterol, this pathway produces multiple metabolic intermediates including farnesyl-pyrophsophate and geranyl-geranyl-phosphate, which are required for protein prenylation and the synthesis of heme, ubichinone and dolichol. As fatty acid and cholesterol biosynthesis require large amounts of NADPH, reduction of NADPH levels by p53 reduces the overall activity of these biosynthetic reactions. FABP = fatty acid binding protein; FATPs = fatty acid transport proteins; CD36 = Thrombospondin receptor/fatty acid translocase; ME = malic enzyme; ELOVL = fatty acid elongase; SCD = stearoyl-CoA desaturase; HMGCS1 = HMG-CoA synthetase 1; HMGCR = HMG-CoA reductase; MVK = mevalonate kinase; PMVK = phosphomevalonate kinase; MVD = mevalonate diphosphate decarboxylase; FDPS = farnesyl diphosphate synthase; IDI = isopentenyl-diphosphate delta isomerase; FDFT1 = Farnesyl-Diphosphate Farnesyltransferase 1; SQLE = squalene synthase; LSS = lanosterol synthase; PANK1 = pantothenate kinase-1; AMPK = AMP activated protein kinase; ACACA = acetyl-CoA carboxylase 1; ACACB = acetyl-CoA carboxylase 2; FASN = fatty acid synthase; ETC = electron transport chain; TCA = tricarboxylic acid cycle.

**Figure 3:**
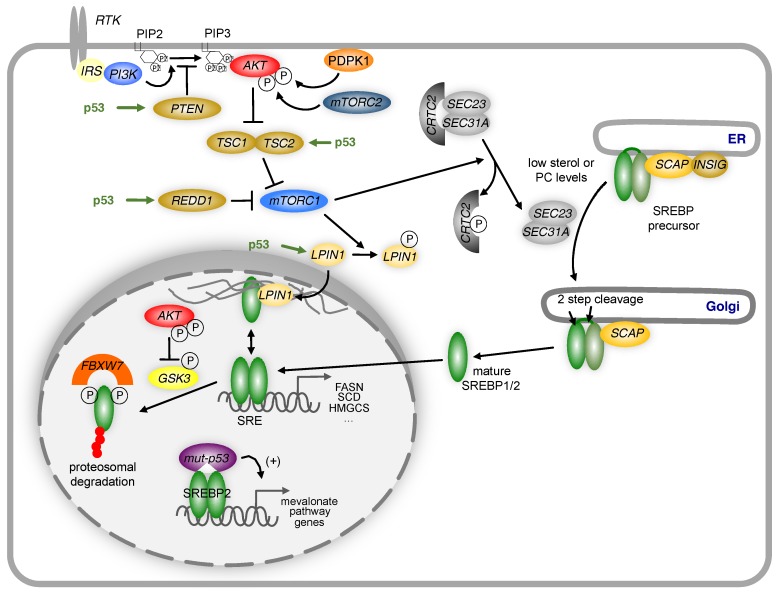
Regulation of SREBP by p53.

Many anabolic processes, including fatty acid biosynthesis, are controlled by a cellular signalling axis involving the complex 1 mechanistic target of rapamycin (mTORC1) [96]. Several negative regulators of mTORC1, including the insulin like growth factor binding protein 1 (IGFBP1), the phosphatase and tensin homolog (PTEN), the tuberous sclerosis protein 2 (TSC2) and the DNA damage inducible transcript 4 (DDIT4/REDD1) [95,97] are also transcriptional targets of p53. Inhibition of the mTORC1 pathway reduces the activity of the sterol regulatory elements binding proteins (SREBPs), a family of basic helix-loop helix transcription factors that control the expression of most enzymes involved in fatty acid and cholesterol biosynthesis [98,99] (Figure 3). While SREBP is activated through the canonical pathway by low cellular sterol levels, mTORC1 dependent activation involves multiple different mechanisms (reviewed in [96]), including enhanced ER/Golgi translocation [100] and control of its sub-nuclear localisation by the phosphatidate phosphatase lipin 1 (LPIN1) [101]. The stability of nuclear SREBP is controlled by ubiquitin-mediated protein degradation through a mechanism involving phosphorylation of SREBP by the glycogen synthase kinases (GSK3) and binding of the F-Box and WD repeat domain containing 7 protein (FBXW7), a subunit of the SCF ubiquitin ligase, to a phosphodegron motif located in the C-terminal part of the mature protein [102]. As the activity of GSK3 is controlled by Akt-dependent phosphorylation, negative regulation of Akt signalling by p53 will result in increased degradation of mature SREBP and reduction in the expression of lipogenic enzymes.

The activity of SREBPs is regulated by p53 through multiple mechanisms. Induction of *PTEN*, *TSC2* or *REDD1* by p53 reduces the activity of the PI3-kinase/Akt/mTORC1 signalling axis. This prevents the translocation of the SREBP precursor from the endoplasmic reticulum (ER) to the Golgi by blocking phosphorylation of the CREB regulated transcription coactivator 2 (CRTC2), thereby inhibiting the activity of the COP II vesicle components SEC23 and SEC31A. In the nucleus, mature SREBP1 can be sequestered to the nuclear periphery through association with LPIN1, unless LPIN1 is phosphorylated by mTORC1 and excluded from the nucleus. Mature SREBP is phosphorylated by GSK3, which promotes recognition by the FBXW7-containing ubiquitin ligase complex SCF (SKP1-cullin-F-box) and targeted for degradation. P53 increases GSK3 activity by limiting Akt activity through the mechanisms mentioned above. Mutant p53 can also directly stimulate the expression of genes in the mevalonate pathway by binding to mature SREBP2 and enhancing promoter transactivation. IRS = Insulin receptor substrate; PIP2 = phosphatidylinositol 4,5-bisphosphate; PIP3 = phosphatidylinositol (3,4,5)-trisphosphate; PDPK1 = 3-Phosphoinositide Dependent Protein Kinase 1, mTORC2 = mechanistic target of rapamycin complex 2.

Moreover, p53 also exerts direct effects on the SREBP pathway. Experiments in a genetic model of obesity (ob/ob mice) showed that disruption of p53 induces lipogenesis in adipocytes by increasing the expression of SREBP1c [103]. Moreover, it has been shown that mutant forms of the p53 protein found in cancer disrupt breast tissue architecture by binding to SREBP2 on chromatin and increasing the expression of SREBP2 target genes [104]. However, this function is absent from wild type p53. Binding of mutant p53 to SREBP2 promotes the generation of geranyl-geranyl-pyrophosphate (GGPP), an intermediate of the cholesterol biosynthesis pathway required for the post-translation modification of Rho-GTPases. Through this mechanism, mutant p53 can promote cell motility and invasion, which are hallmarks of cell transformation and tumour development [105].

In contrast to its negative effect on fatty acid synthesis, p53 increases fatty acid β-oxidation, for example after nutrient deprivation via induction of *AMPK* [106] or through induction of *LPIN1* expression in response to glucose deprivation, oxidative stress or DNA damage [107]. Moreover, in response to glucose deprivation, p53 binds to the PPARG coactivator 1 alpha (PGC1A) and induces the expression of genes promoting cell cycle arrest and ROS clearance [108]. Considering that PGC1A also controls the expression of genes involved in mitochondrial oxidative metabolism [109], the interaction between p53 and PGC1α could also promote fatty acid oxidation. Likewise, it was shown that carnitin palmitoyl transferase 1C (CPT1C) is important to compensate for the adverse effects of glucose deprivation and hypoxia by increasing the levels of fatty acid oxidation and ATP production [110]. CPT1C was also identified as a direct transcriptional target of p53 both in vitro and in vivo [111]. Finally, p53 increases the levels of pantothenate kinase-1 (PANK1), which controls cellular amounts of coenzyme A and could promote the activation of fatty acids for degradation [110]. Increased fatty acid oxidation induced by p53 can also affect other metabolic pathways in cancer cells through negative feedback regulation. Increased production of mitochondrial acetyl-CoA from fatty acids blocks the activity of PDH, the enzyme that controls the entry of pyruvate into the TCA-cycle. Moreover, increased production of citrate from mitochondrial acetyl-CoA leads to the inhibition of PFK1 through allosteric regulation. Therefore, it can be concluded that metabolic regulation by p53 needs to be studied in the context of the complex interactions within the metabolic network [112].

## 7. Roles of p53 in the Tumour Microenvironment

Cancer cells within solid tumours are exposed to oxygen and nutrient gradients that are caused by inefficient vascularisation. Moreover, the complex metabolic interactions between different cell types within the tumour tissue also shape the chemical composition of the tumour microenvironment. In this context, non-cell-autonomous functions of p53 becomes important in regulating the metabolic interaction of cancer cells.

Several studies showed that p53 deletion induces changes in the tumour microenvironment to enhance cancer cell invasion and metastasis formation during tumour progression. This involves blocking the production of secreted factors by macrophages [113], inhibition of the NF-κB-induced inflammatory phenotype and the induction of epithelial to mesenchymal transition (EMT) [114]. For example, p53 induces the degradation of the EMT transcription factor Slug (*SNAI2*) via MDM2 in non-small cell lung cancer [115]. Furthermore, mutant p53 has been also shown to be an important regulator in migration and invasion by affecting signalling by integrins and the EGF receptor (EGFR) [116].

Another important factor of the tumour microenvironment is the availability of oxygen and nutrients provided by the blood stream. The nutrient poor conditions found in insufficiently vascularised tumour areas alter the metabolism of cancer cells in a p53-dependent manner. It has been shown, for example, that glucose deprivation triggers cell cycle arrest by inducing AMPK-dependent phosphorylation and activation of p53 [117]. Thus, p53 participates in an important metabolic checkpoint that blocks proliferation in the absence of sufficient nutrient availability. Moreover, p53 is also involved in shaping the tumour microenvironment by modulating the formation of new blood vessels. It has been shown that p53 blocks tumour angiogenesis by inducing the expression of thrombospondin 1 (*TSP1*) in fibroblasts derived from Li-Fraumeni patients, a hereditary disorder caused by germline mutation of p53 [118]. Likewise, semaphorin 3F (SEMA3F), a direct transcriptional target of p53, was found to inhibit angiogenesis in colon cancer [119]. However, in the absence of the retinoblastoma tumour suppressor protein (pRB), p53 can induce angiogenesis by binding to the promoter of the vascular endothelial growth factor (*VEGF*) gene [120].

## 8. Immune-Regulatory Functions of p53

Increasing evidence indicates that p53 also regulates important functions of the immune system, which is especially relevant in cancer [121]. One important factor is the induction of pro-inflammatory pathways in response to p53 inactivation, which promote tumourigenesis and cancer progression [122,123,124,125]. Interestingly, tumour clearance in response to acute restoration of p53 in lymphomas is associated with induction of apoptosis [126,127]. In contrast, restoration of p53 expression in sarcomas or in a mosaic mouse model of liver cancer resulted in the induction of senescence in cancer cells and activation of the innate immune response by inflammatory cytokines [127,128]. During chronic liver damage, ablation of a p53-dependent induction of senescence in hepatic stellate cells can promote transformation and tumour formation by adjacent epithelial cells, confirming non-cell autonomous tumour suppressor functions of p53 [113]. In addition, oncogene induced senescence leads to the activation of PDH and increased oxidation of pyruvate by the TCA cycle [129]. However, the induction of PDH activity in senescent cells seems to be independent of p53 as depletion of p53 by RNA interference was not sufficient to induce tumour formation in B-Raf^V600E^ transformed melanocytes [129].

Effective tumour clearance by the immune system requires the function of cytotoxic T-cells and there is increasing evidence that T-cells undergo substantial metabolic reprogramming during activation to support their function [130]. Moreover, cancer cells and immune cells can compete for essential nutrients within the microenvironment through metabolic competition. This was demonstrated for cancers in which high glucose uptake was induced by over-expression of hexokinase 2 (HK2) or the MYC oncogene [131]. Therefore, restoration of p53 function could promote T cell activity by reducing the consumption of glucose by cancer cells, thus preventing metabolic competition. The insight into the metabolic activity of different immune cell populations indicates that the complex metabolic interactions between cancer and immune cells have to be considered for the development of therapeutic strategies.

## 9. Targeting Metabolism for the Treatment of p53 Deficient Cancer Cells

Loss or mutation of p53 is a major cause for the resistance to ionising radiation or DNA-damaging drugs in cancer. Therefore, restoring p53 function in an established tumour should provide a major therapeutic benefit [132]. Therapeutic strategies targeting p53 have so far concentrated on two major concepts. The first one aims at tumours that are genetically wild-type for p53 but have lost the expression of the protein due to overexpression or amplification of MDM2 or related proteins. The most prominent member of this class of compounds is the MDM2 inhibitor nutlin (RG7112), which occupies the p53-binding pocket on the surface of the MDM2 protein, leading to stabilisation of the p53 protein [133]. Another strategy is to restore structure of DNA binding domain of mutant p53 by compounds that act as molecular chaperones [134]. One of these compounds, PRIMA-1^MET^ (APR-246), has been shown to efficiently inhibit the growth of p53 mutant tumours [135]. Both nutlin and PRIMA-1^MET^ are currently in clinical evaluation, mainly in combination with DNA damaging chemotherapeutic agents.

Given the importance of metabolic reprogramming in cancer, it is a reasonable expectation that metabolic processes can also be targeted directly for cancer therapy. However, inhibition of anabolic pathways, such as protein and lipid biosynthesis, limits the ability of cancer cells to grow and proliferate. Targeting these processes would most likely induce tumour stasis, which is often not sufficient for stable disease control. It is therefore essential to identify those metabolic vulnerabilities in cancer cells that, when targeted, induce cancer cell death to achieve lasting therapy response.

As p53 is part of a metabolic checkpoint that is activated by nutrient starvation, loss of p53 renders cancer cells more sensitive to limited nutrient supply [59,106]. Under these conditions, p53 deficient cancer cells fail to downregulate energy demanding biosynthetic processes. This leads to further depletion of essential nutrients and often results in increased oxidative stress and cell death. Identifying those nutrients and metabolic processes that function in a synthetic lethal manner with loss or mutation of p53 could reveal novel avenues for the selective targeting of p53-deficient cancer cells. In this context, large scale screening approaches that investigate the contribution of nutrient transporters could be particularly useful [136]. One candidate drug that could exploit the loss of p53-dependent metabolic checkpoint control is metformin, a biguanide used in the management of diabetes. Indeed, metformin and the related drug phenformin are currently undergoing extensive clinical testing either alone or in combination with established cancer therapeutics.

Another important function of p53 in metabolic regulation is the control of cellular NADPH production. As NADPH is required for many biosynthetic processes, targeting the synthesis and regeneration of this cofactor should have a global effect on cellular metabolic activity. Moreover, the dual function of NADPH in macromolecule synthesis and antioxidant production makes it a particularly attractive target. Reduction of NADPH levels would not only block the ability of cancer cells to proliferate but also induce oxidative damage and cell death. Indeed, the anti-cancer agent RRx-001, which is undergoing clinical trials in several cancer types, may exert its anti-proliferative effects through inhibition of G6PD [137].

Altered lipid metabolism could also be a therapeutic target in p53 deficient cancers. Inhibition of fatty acid biosynthesis, for example by the FASN inhibitor TVP-2647, is currently tested in solid tumours [92]. In addition, statins, a class of cholesterol-lowering drugs that block a key enzyme of the mevalonate pathway, could be particularly efficient in p53 mutant cancers [138]. However, the important role of p53 in metabolic control may also complicate treatment strategies that aim to reactivate p53 in tumours. Restoring p53 function may actually promote the survival of cancer cells under certain conditions, such as in nutrient poor tumour areas. This could lead to therapy resistance, for example in combination with anti-angiogenic drugs. A comprehensive understanding of the metabolic functions of p53 is therefore crucial to develop successful strategies for cancer treatment.
